# Highly Dynamic Microtubules Improve the Effectiveness of Early Stages of Human Influenza A/NWS/33 Virus Infection in LLC-MK2 Cells

**DOI:** 10.1371/journal.pone.0041207

**Published:** 2012-07-20

**Authors:** Flora De Conto, Enrica Di Lonardo, Maria Cristina Arcangeletti, Carlo Chezzi, Maria Cristina Medici, Adriana Calderaro

**Affiliations:** Section of Microbiology, Department of Pathology and Laboratory Medicine, University of Parma, Parma, Italy; Johns Hopkins University - Bloomberg School of Public Health, United States of America

## Abstract

**Background:**

This study aims to investigate the role of microtubule dynamics in the initiation of NWS/33 human influenza A (NWS) virus infection in MDCK and LLC-MK2 mammalian kidney cells. We previously demonstrated a host-dependent role of the actin cytoskeleton in inducing restriction during the early phases of NWS infection. Furthermore, we showed the differential infectious entry of NWS virus in the above mentioned cell models.

**Methodology/Principal Findings:**

By first employing a panel of microtubule-modulators, we evidenced that microtubule-stabilization negatively interferes with NWS replication in LLC-MK2 but not in MDCK cells. Conversely, microtubule-depolymerization improves NWS growth in LLC-MK2 but not in the MDCK model. By using immunofluorescence labelling and Western blotting analyses upon NWS infection in mammalian kidney cells, it was observed that the occurrence of alpha-tubulin hyperacetylation - a post-translational modified form suggestive of stable microtubules - was significantly delayed in LLC-MK2 when compared to MDCK cells. Furthermore, mock-infected LLC-MK2 cells were shown to have higher levels of both acetylated alpha-tubulin and microtubule-associated protein 4 (MAP4), the latter being essential for the maintenance of normal microtubule polymer levels in interphase epithelial cells. Finally, to obtain highly dynamic microtubules in LLC-MK2 cells, we knocked down the expression of MAP4 by using a RNA-mediated RNA interference approach. The results evidenced that MAP4 silencing improves NWS growth in LLC-MK2 cells.

**Conclusion:**

By evidencing the cell type-dependent regulatory role of microtubule dynamics on NWS replication in mammalian kidney cells, we demonstrated that microtubule-stabilization represents a restriction factor for the initiation of NWS infection in LLC-MK2 but not in MDCK cells.

## Introduction

The microtubule (MT) cytoskeleton is a highly regulated system involved in many cellular functions, such as endocytic/exocytic vesicles transport, spindle assembly and signalling pathways compartmentalization [1]–[3].

MTs are hollow tubes made up by the association of several protofilaments composed of alternating alpha– and beta–tubulin monomers. MTs have a distinct structural polarity, and possess special dynamic properties. The so-called “dynamic instability” of MTs - which may vary among cell types and phases of the cell cycle - is a direct consequence of stochastic alternations between periods of catastrophe (growth to shortening) and rescue (shortening to growth). Moreover, it has been suggested that several proteins moving along the MT lattice are directly involved in the regulation of MT dynamics. Among these regulating factors, microtubule-associated proteins (MAPs), such as tau, MAP2, and MAP4, by interacting with the acidic C-terminal region of tubulin, polymerize and stabilize MTs [1], [4], [5]. On the other hand, the stathmin/oncoprotein 18, by expressing catastrophe-promoting activities, prevents MT-assembly [4], [6], [7].

Increasing evidences support the notion that the functional diversity of MTs is also dependent on several post-translational modifications, such as tyrosination, glutamylation and acetylation [2]. Among these events, the acetylation of the epsilon-amino group of alpha-tubulin lysine 40 is conceived as a major regulator of cell signalling, differentiation and survival [2], [8]. Moreover, acetylated alpha-tubulin is a well recognized marker of stable MTs [9]. In this regard, it has been suggested that the MT-stabilizing drug paclitaxel (taxol) promotes alpha-tubulin acetylation [8]. Very important, the balance between acetylation and deacetylation conditions of tubulin regulates MT functions [10].

The intracellular transport mediated by MTs is bidirectional and involves plus- and minus-end directed molecular motors belonging to dynein and kinesin families [11], [12]. Viruses have found elegant solutions to hijack MT-associated motors by direct interaction or inside membranous compartments [13]–[15]. Interestingly, heterodimers of alpha- and beta-tubulin were found inside virions [16]–[18].

Concerning specifically influenza viruses, it has been shown that viral ribonucleoproteins co-localize with MTs [19] and viral RNA polymerases interact with beta-tubulin [20]. Furthermore, influenza ribonucleoproteins accumulate near the microtubule organizing centre in the course of their cytoplasmic transport [21]. Yet again, Momose *et al.* [22] evidenced that in polarized epithelial cells the trafficking of influenza ribonucleoproteins to the apical plasma membrane requires the targeting to the endoplasmic reticulum and occurs along MTs. Accordingly, Husain and Harrod [23] showed that influenza A virus infection in epithelial cells enhances the levels of acetylated alpha-tubulin in order to favour the polarized transport of viral components during assembly. Fascinatingly, recent data [24] highlight the differential regulatory role of specific Class I histone deacetylases on both endosome maturation and influenza A virus infection progression, thus evidencing their relationship with MT cytoskeleton organization and centrosome cohesion.

We previously demonstrated a host-dependent role of the actin cytoskeleton in inducing restriction during the early phases of human influenza A/NWS/33 virus infection in mammalian kidney cells [25]. Moreover, we showed the differential infectious entry of NWS virus in mammalian kidney cells [26]. Our present work focuses on the role of MT dynamics in the initiation of the NWS life cycle in LLC-MK2 and MDCK cells, with different susceptibility upon infection. Here we first examined the effects induced on NWS infection by drug treatments of MTs, and then we explored the involvement of post-translational events acting on MT-stabilization. Finally, by using a RNA-mediated RNA interference approach, we investigated the role of MAP4, which is essential for the maintenance of normal MT polymer levels in interphase epithelial cells.

## Materials and Methods

### Cells and viruses

Madin-Darby canine- (MDCK), rhesus monkey- (LLC-MK2), and newborn swine- (NSK) kidney cell lines from the “Istituto Zooprofilattico Sperimentale della Lombardia e dell'Emilia-Romagna” (Brescia, Italy) were propagated in Earle's modified Minimum Essential Medium (E-MEM), supplemented with 2 mM L-glutamine, antibiotics (100 U/ml penicillin, 100 µg/ml streptomycin), and 1% or 10% foetal bovine serum. Culture media and supplements were from PAA (The cell culture company).

The NWS/33 strain of human influenza A virus (subtype H1N1; ATCC VR 219) was propagated as previously detailed [25]. The Mallard/Italy/303394-11/03 strain of avian influenza A (Mallard/03) virus (subtype H1N1; “Istituto Zooprofilattico Sperimentale della Lombardia e dell'Emilia Romagna” – Parma, Italy) was used as reference in some experiments. The latter strain was propagated for 96 h at 37°C in the allantoic cavities of 11-day-old fertilized hen eggs. The harvested allantoic fluid was then centrifuged for 30 min at 12,096× g before viral yield evaluation by plaque assay. Virus stocks were stored at −80°C until further use.

MDCK, LLC-MK2, and NSK cells grown to confluence in shell-vials or 6-well plates were infected with NWS virus at a multiplicity of infection (m.o.i.) of 0.1 plaque forming units (p.f.u.)/cell in order to show up their differential susceptibility upon infection. On the other hand, for both NWS and Mallard/03 viruses, a m.o.i. of 1 or 10 p.f.u./cell was also employed in order to infect all the cells of the monolayer, thus assessing more accurately specific features of the examined infectious cycle. After absorption for 75 min at 4°C, the viral inoculum was removed and cells were incubated for the planned infectious time.

### Antibodies and drugs

Primary antibodies used for indirect immunofluorescence (IIF) assays included mouse monoclonal antibodies to viral nucleoprotein (NP) (Argene; dilution 1∶30), beta-tubulin (Boehringer Mannheim; dilution 1∶50) and acetylated alpha-tubulin (Santa Cruz Biotechnology; dilution 1∶50). Bound antibodies were detected by Alexa Fluor 568 or Alexa Fluor 488 goat anti-mouse IgG antibodies (Molecular Probes; dilution 1∶500).

Primary antibodies used for Western blotting included rabbit polyclonal anti-MAP4 (Millipore; dilution 1∶1,400), mouse monoclonal anti-acetylated alpha-tubulin (dilution 1∶200), mouse monoclonal anti-beta-tubulin (dilution 1∶200), and sheep polyclonal anti-hemagglutinin (HA) (National Institute for Biological Standards and Control, UK; dilution 1∶90) antibodies. Bound antibodies were, respectively, detected by alkaline phosphatase-conjugated anti-rabbit (Dako; dilution 1∶300), anti-mouse (Dako; dilution 1∶300), and anti-sheep (Sigma-Aldrich; dilution 1∶7,500) antibodies.

The MT-modulators nocodazole (NOC; LLC-MK2, MDCK: 2 µM; NSK: 1 µM), paclitaxel (TAX; LLC-MK2, MDCK: 2 µM; NSK: 1 µM) and sodium orthovanadate (Na_3_VO_4_, 50 µM) were added to cell monolayers for 30 min at 37°C. Following either a mock or a given drug treatment, the cells were infected with NWS virus in the presence or absence of drugs. Next, the viral inoculum was removed and cells were incubated in E-MEM containing the drugs. At 2 h post-infection (p.i.) the cells were either washed and then maintained in drug-free culture medium (early treatment) or kept in drug-containing medium for the entire infectious period. Conversely, for late p.i. analyses, drugs were added at 5 h p.i., assuming that the very early stages of NWS infection had already been concluded, and then maintained for the planned infectious time. NOC and TAX were obtained from Calbiochem. Na_3_VO_4_ was purchased from Sigma-Aldrich. Drugs were each reconstituted and stored according to the manufacturers' instructions and used with matching concentrations of the vehicle in the cell control. The highest applicable concentration of each compound was preliminary assessed by chromatin staining with 4′,6-diamidino-2-phenylindole dihydrochloride (Sigma-Aldrich) in order to avoid any cytotoxic effect. Moreover, cell viability was assessed by trypan blue staining.

### Indirect immunofluorescence assay

Uninfected and infected mammalian kidney cells were fixed with chilled methyl alcohol at −20°C for 10 min. Alternatively, for the analysis of the MT cytoskeleton, cells were washed once at 37°C with the cytoskeleton buffer [10 mM 1.4-piperazine-bis-ethane sulfonic acid (pH 6.9), 100 mM NaCl, 3 mM MgCl_2_, 300 mM sucrose, 3 mM ethylene glycol tetraacetic acid, 1 mM phenylmethanesulfonylfluoride] and then permeabilized and simultaneously fixed with a mixture of 0.7% Triton X-100 and 1% paraformaldehyde in the cytoskeleton buffer for 20 min at 37°C. Following residual aldehyde quenching by incubation with 1% bovine serum albumin in phosphate-buffered saline (PBS: 7 mM Na_2_HPO_4_, 1.5 mM KH_2_PO_4_, 137 mM NaCl, 2.7 mM KCl; pH 7.4), the immunoreaction was carried out with primary antibodies, applied for 1 h at 37°C. Next, the cells were washed twice in PBS, and secondary antibodies were applied for 45 min at 37°C. For negative controls, the primary antibodies were replaced by 0.2% bovine serum albumin in PBS. For NP fluorescence quantization, ten randomly selected fields per cell monolayer were counted, and positive cells expressed as mean values percentage of the total cell number per field evaluated by chromatin staining of nuclei. Finally, the cells were mounted in buffered glycerol solution (Argene) and analyzed by an epifluorescence microscope (DMLB Leica). Chemicals were from Sigma-Aldrich.

### Microtubule isolation and high-salt extraction assays

MT isolation and high-salt extraction assays were carried out as described in Kannan *et al.* [27], with some modifications. Briefly, LLC-MK2 cells grown in 6-well plates were infected with NWS virus at a m.o.i. of 1 for 24 h, then cell lysates were collected with a cell scraper in the MT-stabilizing (PEM) buffer [100 mM 1.4-piperazine-bis-ethane sulfonic acid (pH 6.9), 5 mM MgCl_2_, 1 mM ethylene glycol tetraacetic acid] supplemented with 40 µM TAX. Samples were then transferred into microcentrifuge tubes before homogenization with a syringe and 25-gauge needle. Following a 10-min incubation at 37°C, samples were centrifuged at 100,000× g for 30 min at 37°C. The obtained supernatant fractions were stored, and corresponding pellets homogenized in PEM or PEMS (PEM buffer plus 500 mM KCl) buffers before centrifugation at 100,000× g for 30 min at 37°C. The resulting pellets (samples P) were then treated in Laemmli buffer [25% glycerol, 0.1% bromophenol blue, 2% sodium dodecyl sulfate, 100 mM dithiothreitol, 50 mM Tris–HCl (pH 6.8)]. The corresponding supernatant fractions were first pooled with the previously obtained supernatants and then subjected to protein precipitation with 10% trichloroacetic acid, before treatment in Laemmli buffer (samples S). Finally, all samples were heated at 95°C for 5 min before Western blotting analysis.

### Western blotting analysis

LLC-MK2 and MDCK cells grown in 6-well plates were mock-infected or infected with NWS or Mallard/03 viruses at a m.o.i. of 1 for, respectively, 24 or 48 h. Cell lysates were then collected as previously described [26]. Equal amounts of proteins (30 µg/lane) were resolved by sodium dodecyl sulphate-8% (MAP4) or -12.5% (HA, beta–tubulin, and acetylated alpha–tubulin) polyacrylamide gel electrophoresis, and then transferred onto nitrocellulose membranes. After a blocking-step in PBS containing 0.2% Tween-20 supplemented with 4% non-fat dry milk (PBST) for 1 h at room temperature, the membranes were incubated for 1 h in PBST with primary antibodies. After three washing steps in PBST, the membranes were then incubated for 1 h in PBST containing alkaline phosphatase-conjugated antibodies. Thereafter, the membranes were washed in PBST and bound antibodies detected with 5-bromo-4-chloro-3-indolyl-phosphate/nitro blue tetrazolium buffered substrate (Sigma-Aldrich).

### Knockdown of MAP4

To knock down MAP4 in LLC-MK2 cells, two 21-mer small interfering RNAs (siRNAs) duplexes (MAP4-1: GGGATTAGTTAGAACCTTA; MAP4-2: GTTCCAAGGACAATATTAA) targeting the MAP4 gene were obtained from Sigma-Aldrich. LLC-MK2 cells at 75% confluence in shell-vials were transfected with 20 nM of either MAP4 gene-specific siRNAs or control siRNA (21-mer non effective scrambled double-stranded siRNA), by using the N-TER™ Nanoparticle siRNA Transfection System (Sigma-Aldrich). To verify the efficacy of MAP4 silencing, whole lysates of transfected cells were analyzed by Western blotting assays at different times post-transfection. Initial experiments revealed that the use of the MAP4-1 duplex for 48 h resulted in the most significant down-regulation of MAP4. Hence, the MAP4-1 construct was chosen for further studies. Untransfected and 48 h-transfected LLC-MK2 cells were then infected with NWS virus at a m.o.i. of 0.1 for 24 h. Finally, NP-positive cells were quantized by IIF staining and viral yields from culture supernatants determined by fifty % tissue culture infectious dose (TCID_50_) assays.

### TCID_50_ assay

To evaluate viral yields, 10-fold serial dilutions of culture supernatants from infected kidney cells were prepared in serum-free E-MEM. To this aim, 100 µl of diluted supernatants were added to each well of a 96-well microplate (4 replicates per dilution), then 100 µl of a freshly prepared MDCK cellular suspension (cell density: 2.16×10^5^ cells/ml in E-MEM supplemented with 2.5% decomplemented foetal bovine serum) were overloaded. Viral yields were evaluated after 4 days of incubation at 37°C and expressed as log_10_ of TCID_50_ values per 100 µl of culture supernatant.

## Results

### Microtubule-perturbing drugs alter the morphology of the microtubule cytoskeleton in LLC-MK2 and MDCK cells

**Figure 1:**
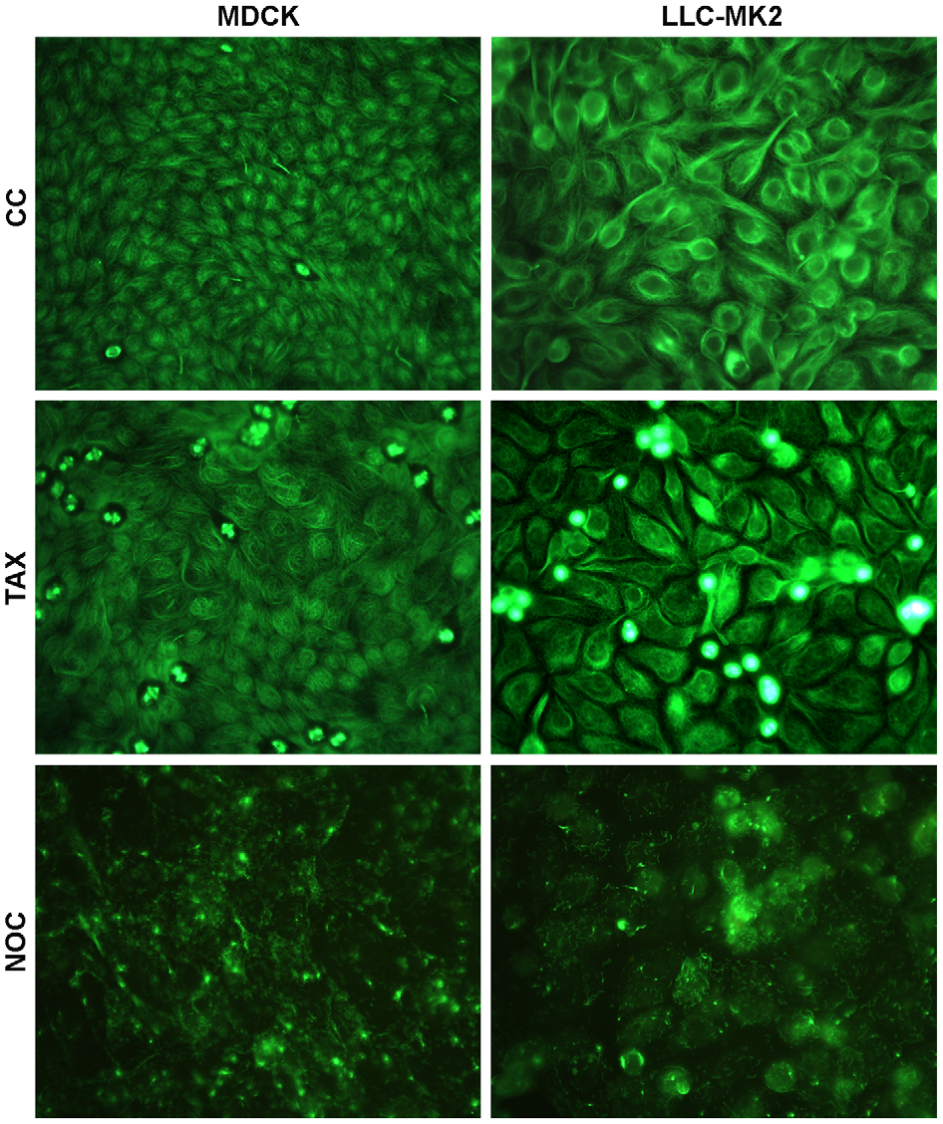
Effects of MT-perturbing drugs on the microtubular cytoskeleton morphology in kidney cells. LLC-MK2 and MDCK cells were either untreated (CC) or subjected to TAX or NOC treatments for 3 h 45 min, before IIF assays with anti-beta-tubulin antibodies. Pictures were collected by using a conventional fluorescence microscope (magnification: 500×). Essentially similar results were obtained in two independent experiments.

The effect of drugs that either depolymerize (NOC) or stabilize (TAX) the MTs was first assessed in the course of IIF assays carried out in MDCK and LLC-MK2 cells in order to analyze the MT cytoskeleton morphology. To this purpose, kidney cells were either untreated or subjected to NOC and TAX treatments for a period of 3 h 45 min, before IIF evaluation of the beta-tubulin pattern (Figure 1). By examining the effects of NOC treatments in both cell models, we assessed the disruption of the filamentous MT network of untreated cells. Conversely, TAX treatments did not result in robust changes in the cell shape or MT network. However, the appearance of compact bundles of MTs not evidenced in mock-treated cells was indicative of TAX-induced stabilization of pre-existing MT polymers, thereby preventing MT-depolymerization. Moreover, the detection of several mitotic spindles also supported the suppression of MT dynamics.

### Microtubule dynamics differentially modulate the replication cycle of influenza A/NWS/33 virus in mammalian kidney cells

To investigate the role of the MT cytoskeleton during the NWS virus replication cycle in LLC-MK2 and MDCK cells, we then examined the effects induced by the above mentioned MT-modulators. To this purpose, cells were either untreated or pretreated with NOC or TAX, and then infected for 24 h with NWS virus (m.o.i. = 0.1 p.f.u./cell) in the absence or presence of drugs. NOC and TAX were either kept in the culture media throughout the infectious period or withdrawn at 2 h p.i.. Alternatively, untreated cells were first infected, then drugs were added at 5 h p.i. without further removal. Untreated and infected controls were examined in parallel. Infection progression was monitored by IIF staining of NP-positive cells.

**Figure 2:**
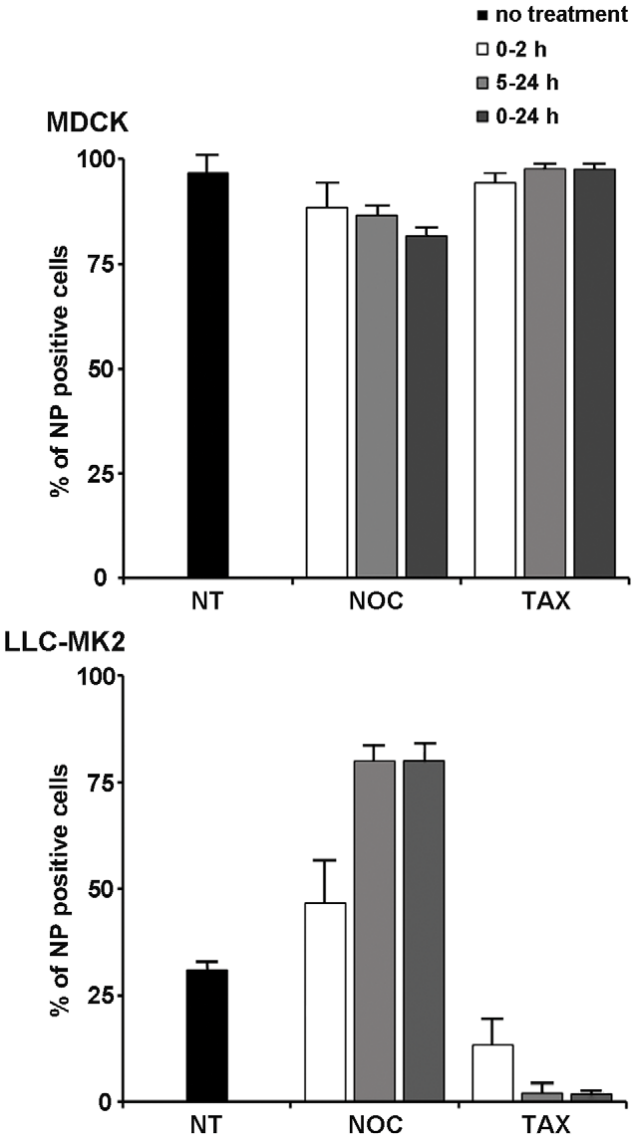
MT-altering compounds exert cell type-dependent effects on influenza A/NWS/33 virus growth in mammalian kidney cells. MDCK and LLC-MK2 cells were mock-treated (NT) or pretreated with NOC or TAX for 30 min (0–2 h; 0–24 h), and subsequently inoculated with NWS virus (m.o.i. = 0.1 p.f.u./cell; 24 h) in the absence (NT) or presence of drugs. NOC and TAX were either kept in the culture media for 2 h p.i. and then withdrawn (0–2 h) or maintained throughout the entire infectious period (0–24 h). Alternatively, NWS infection was carried out for 5 h in drug-free culture medium, and then the cells were treated with NOC or TAX for the remnant infectious period (5–24 h). Next, the cells were labelled with anti-NP antibodies by IIF. The number of NP-positive cells in relation to total cell population was expressed as a percentage. Each sample was processed in duplicate. Values represent the mean of three independent experiments. Error bars in graphs represent standard deviations.

Figure 2 shows a massive increase of NP levels when NOC was added throughout NWS infection for 24 h in LLC-MK2 cells, whilst a slight decrease was observed in the MDCK model. Similarly, early (0–2 h) and late (5–24 h) treatments with NOC significantly triggered NP levels in LLC-MK2 cells. On the other hand, infection progression was highly impaired upon treatments with TAX in LLC-MK2 but not in MDCK cells. In the latter model, the effects of early and late treatments with both MT-modulators were similar to 24 h-treatments.

Next, we investigated whether MT-altering drugs might interfere with the emergence of the viral progeny by using TCID_50_ assays. The results (not shown) were consistent with IIF analyses.

In order to further analyze the role of MT dynamics during NWS infection, additional experiments were carried out in semi-permissive NSK cells (Figure S1). To this aim, we first assessed the alteration of the MT cytoskeleton induced by NOC and TAX in NSK cells (Figure S1A), and then we analyzed the effects on NWS replication (Figure S1B). Interestingly, the results shown in Figure S1B evidence that both drugs had an adverse effect on NP expression. More specifically, TAX treatments exerted slightly less negative effects in NSK (Figure S1B) when compared to LLC-MK2 cells (Figure 2). Conversely, NOC treatments induced a consistent decrease of NP expression in NSK cells, thus, unlike LLC-MK2, inhibiting NWS growth.

By taking into account that stable MTs had major adverse effects on NWS replication in LLC-MK2 than in NSK cells, we next draw our attention on the former model. The NSK model was not investigated further.

### Both MT-stabilization and dynein inhibition affect the early stages of NWS virus replication in LLC-MK2 cells

Finding that stable MTs interfere with viral replication in LLC-MK2 but not in MDCK cells, we further analyzed the contribution of MT-stabilization in the initiation of the NWS life cycle in the former model. To this aim, the timing of the nuclear delivery of NWS virus was preliminarily assessed in LLC-MK2 cells by experimental infectious assays carried out at a m.o.i. of 10 p.f.u./cell in order to better visualize incoming viruses. The findings (not shown) supported that the nuclear localization of viral ribonucleoproteins starts around 2 h 30 min p.i..

**Figure 3:**
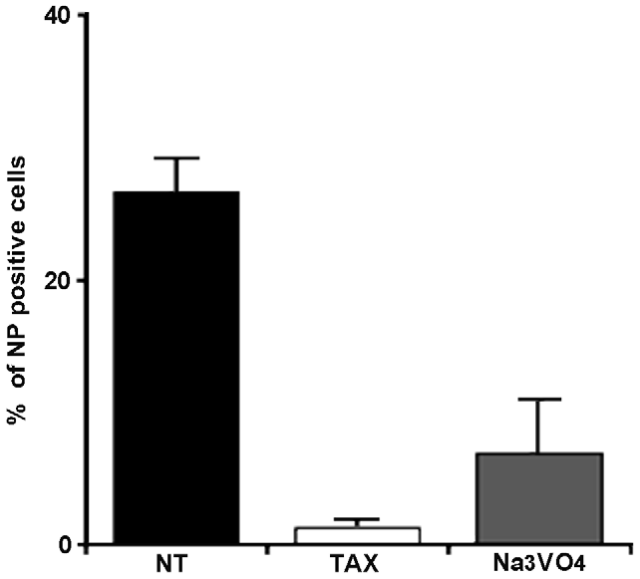
Dynamic MTs are required for the initiation of NWS virus infection in LLC-MK2 cells. LLC-MK2 cells were either untreated (NT) or kept in E-MEM plus TAX or Na_3_VO_4_ for 30 min and then infected for 5 h with NWS virus (m.o.i. = 0.1 p.f.u./cell) in the absence (NT) or presence of drugs. TAX and Na_3_VO_4_ were kept in the culture media for 2 h p.i. and then cell monolayers were incubated in drug-free media for the residual infection time. The graphic represents the percentage of NWS infected cells, according to NP labelling by IIF. Each sample was processed in duplicate. Mean values from three independent experiments were analyzed. Error bars correspond to standard deviations.

Next, to allow a more punctual analysis of TAX-induced effects, LLC-MK2 cells were either untreated or pretreated for 30 min with TAX and then infected for 5 h with NWS virus (m.o.i. = 0.1 p.f.u./cell) in the absence or presence of the drug. In order to target the initiation of NWS infection, TAX was kept in the culture medium for the first two hours p.i. and then withdrawn. Figure 3 shows a dramatic reduction of NP levels in TAX-treated LLC-MK2 cells.

Observing that stable MTs negatively interfere with early phases of NWS growth in LLC-MK2 cells, we were interested to ascertain the involvement of the MT network in the viral cytoplasmic trafficking. To this aim, we examined the role of dynein, a minus-end directed MT-based motor, by using a dynein inhibitor (Na_3_VO_4_) at the same experimental conditions adopted for TAX. Interestingly, the results in Figure 3 demonstrated that although NWS transport was mostly blocked by dynein inhibition, some cytoplasmic trafficking may have still occurred.

### Viral hemagglutinin interacts with MTs upon NWS infection in LLC-MK2 cells

**Figure 4:**
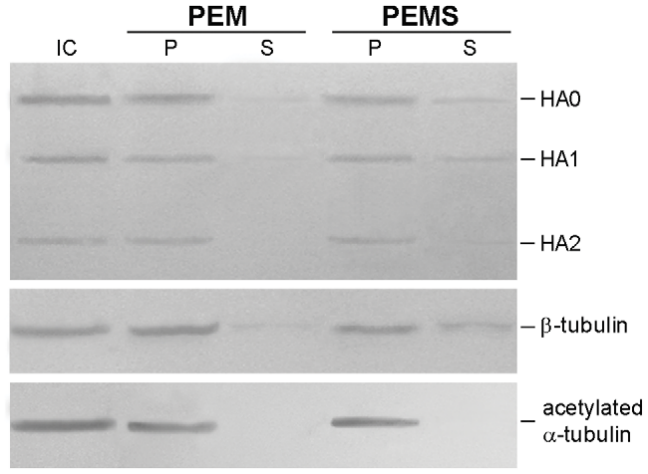
MT isolation assays evidence the interaction between viral HA and MTs in LLC-MK2 cells. LLC-MK2 cells were infected with NWS virus at a m.o.i. of 1 for 24 h, then MT isolation (PEM panel) and high-salt extraction (PEMS panel) assays were carried out as described in the Methods section. Protein extracts from MT-enriched pellets (P lanes) and corresponding supernatants (S lanes) were analyzed by immunoblotting with antibodies to viral HA (shown are the HA0, HA1 and HA2 subunits), beta-tubulin, and acetylated alpha-tubulin. Total cell lysate from infected cells (IC) was included as a control. Essentially similar results were obtained in two independent experiments.

Finding that dynamic MTs positively influence the virus life cycle in LLC-MK2 cells, we next investigated the existence of interactions between the incoming virus and MTs. To this aim, NWS-infected LLC-MK2 cells (m.o.i. = 1 p.f.u./cell; 24 h) were first homogenized and then centrifuged in order to separate the polymerized MT-enriched fraction from tubulin monomers, as detailed in the Methods section. Alternatively, infected cells were treated with a high-salt buffer to promote the release of MAPs. Untreated lysates from infected LLC-MK2 cells were used as controls. Samples were then examined by Western blotting. The results shown in Figure 4 (PEM panel) evidenced that viral HA was mostly found in the pellet fraction, thus suggesting its predominant co-sedimentation with polymerized MTs. Conversely, very small amounts of HA were detected in the corresponding supernatant fraction. Partially different results (Figure 4, PEMS panel) were obtained by high-salt extraction, in that HA signal was more easily detected also in the supernatant fraction. Very important, the presence of acetylated alpha-tubulin in both PEM and PEMS pellets but not in the high-salt supernatant fraction substantiated the stability of the MT-enriched fraction. Although our findings were suggestive of electrostatic interactions between HA and polymerized MTs, cautious conclusions were drawn. We did not investigate further these data.

### Differential activation of post-translational hyperacetylation upon influenza A virus infection in mammalian kidney cells

**Figure 5:**
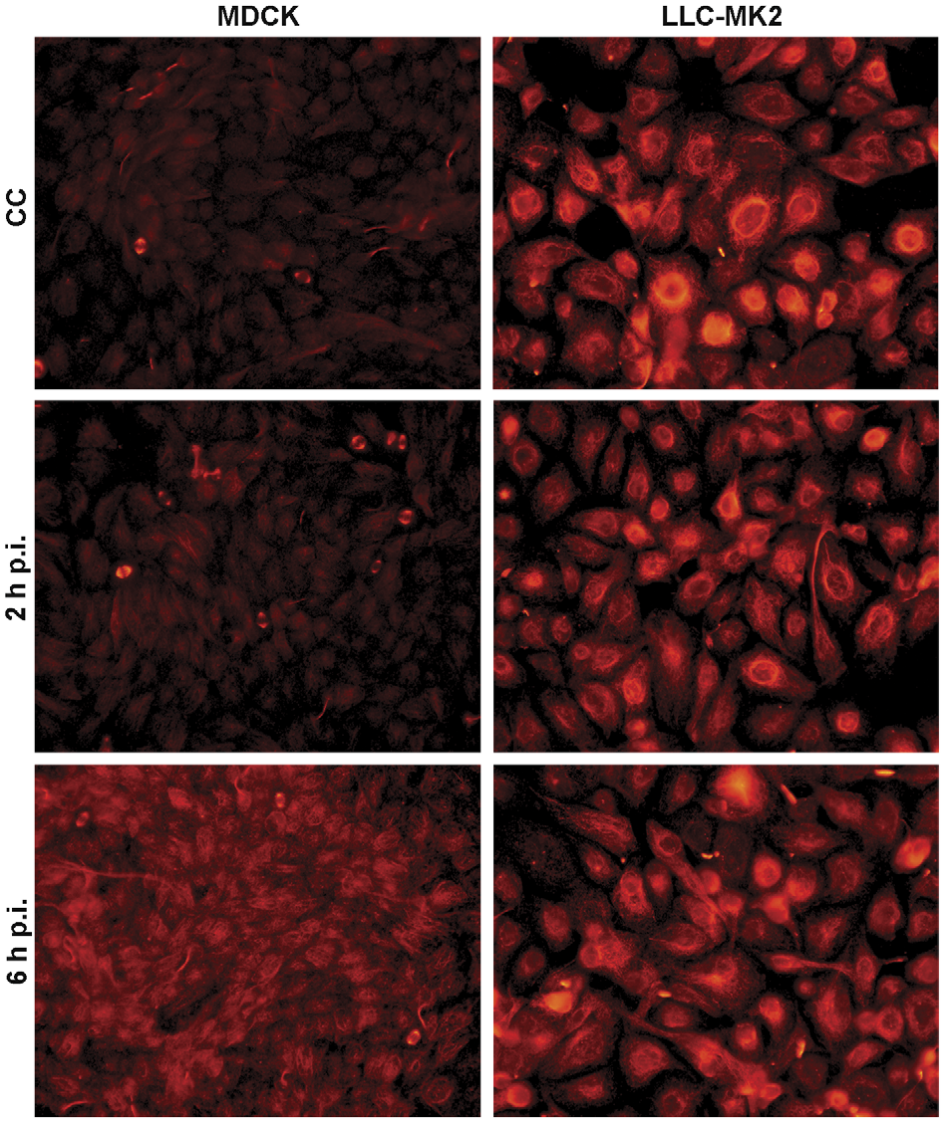
Differential hyperacetylation of alpha-tubulin during a 6 h-replication cycle of NWS virus in mammalian kidney cells. LLC-MK2 and MDCK cells were either uninfected (CC) or subjected to NWS infection (m.o.i. = 1 p.f.u./cell). To this aim, cell monolayers were fixed at each hour p.i. of a 6 h-infectious cycle, before IIF labelling using antibodies against acetylated alpha-tubulin. Shown are mock-infected (CC) and, respectively, 2 h- and 6 h-infected cells, as the most representative images of the obtained results. Pictures were collected by using a conventional fluorescence microscope (magnification: 500×). Each IIF analysis was done in duplicate. Essentially similar results were obtained in three independent experiments.

Post-translational modifications play a critical role in regulating protein functions. Because post-translational tubulin hyperacetylation is a well-recognized marker of stable MTs, we next examined the level of acetylated alpha-tubulin in LLC-MK2 and MDCK cells in order to investigate its relationship with NWS infection. To this purpose, kidney cells were either uninfected or subjected to NWS infection (m.o.i. = 1 p.f.u./cell) and then the IIF pattern of acetylated alpha-tubulin was evaluated at each hour p.i. of a 6 h-infectious cycle. Interestingly, the results shown in Figure 5 demonstrated that mock-infected LLC-MK2 cells, conversely to MDCK, present a huge subpopulation of stable MTs marked by acetylation. Moreover, in both models the levels of acetylated alpha-tubulin appear almost unchanged from 2 (Figure 5) to 5 h p.i. (not shown). Furthermore, while in 6 h-infected LLC-MK2 cells the amount of acetylated alpha-tubulin was still comparable to that of mock-infected cells, a consistent increase in the stable MT subpopulation was observed in the MDCK model.

**Figure 6:**
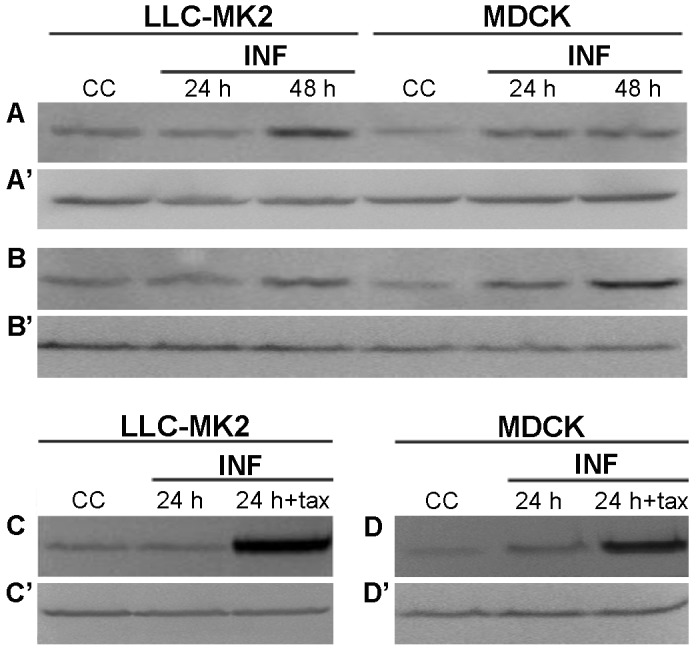
Differential increase of acetylated alpha-tubulin levels upon influenza A virus infection in mammalian kidney cells. (A,A′,B,B′) LLC-MK2 and MDCK cells were infected with (A,A′) NWS or (B,B′) Mallard/03 viruses at a m.o.i. of 1 for 24 or 48 h. (C,C′) LLC-MK2 and (D,D′) MDCK cells were either untreated or pretreated with TAX for 30 min, and then infected with NWS virus (m.o.i. = 1 p.f.u./cell; 24 h) in the absence (24 h) or presence (24 h+TAX) of the drug. (A,B,C,D) Western blotting detection of acetylated alpha-tubulin. (A′,B′,C′,D′) The presence of beta-tubulin was checked in parallel as a protein loading control. Total lysates from uninfected cells (CC) were also examined. Fundamentally similar results were obtained in two independent experiments.

In order to further shed light on alpha-tubulin acetylation kinetics, kidney cells were then infected with NWS virus (m.o.i. = 1 p.f.u./cell) for 24 or 48 h, before Western blotting analyses. As shown in Figure 6A, the increase of tubulin acetylation was significantly delayed in LLC-MK2 cells when compared to MDCK, as in the former model it became evident around 48 h p.i.. With the purpose of investigating the dependence of the observed hyperacetylation pattern from specific features of NWS, we carried out analogous Western blotting analyses by using a low pathogenic avian influenza A virus (Mallard/03), as reference. Interestingly, findings in Figure 6B showed that Mallard/03 behaves very similarly to NWS.

Finally, LLC-MK2 and MDCK cells were either untreated or pretreated for 30 min with TAX and then infected for 24 h with NWS virus (m.o.i. = 1 p.f.u./cell) in the absence or presence of the drug. Importantly, the results in Figure 6C confirmed that TAX was able to put forward the hyperacetylation, when compared to mock-treated and infected LLC-MK2 cells. Moreover, the hyperacetylation level observed in TAX-treated and infected MDCK cells (Figure 6D) was slightly less pronounced than in LLC-MK2 cells, possibly implying that the very low amount of stable MTs present in untreated cells might render less effective the occurrence of further TAX-induced increases.

### Cell type-dependence of MAP4 expression in mock-infected and NWS-infected mammalian kidney cells

**Figure 7:**
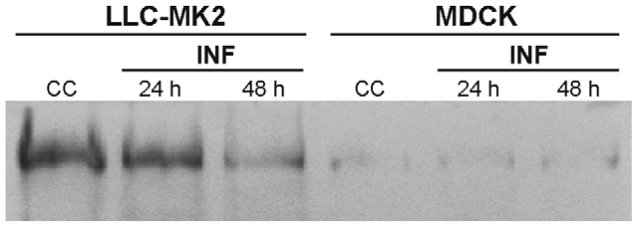
Differential expression of MAP4 in mock-infected and NWS-infected mammalian kidney cells. LLC-MK2 and MDCK cells were infected with NWS virus at a m.o.i. of 1 for 24 h or 48 h, then Western blotting detection of MAP4 was carried out. Total lysates from uninfected cells (CC) were examined in parallel. Essentially similar results were obtained in two independent experiments.

To further shed light on the role of MT-stabilization in modulating NWS infection, we next explored the possible contribution of MAP4, a MT-associated protein showing rescue-promoting activities during the *in vitro* assembly of MTs. To this aim, LLC-MK2 and MDCK cells were either uninfected or infected with NWS virus (m.o.i. = 1 p.f.u./cell) for 24 or 48 h, before Western blotting analyses. The results in Figure 7 evidenced the presence of a huge amount of MAP4 in uninfected LLC-MK2 cells, whilst only faint signals were discernible in the MDCK model. Moreover, upon NWS infection we observed a progressive decrease of MAP4 expression in LLC-MK2 cells, whilst in MDCK cells its poor amount was unchanged.

### MAP4 depletion improves the outcome of NWS infection in LLC-MK2 cells

**Figure 8:**
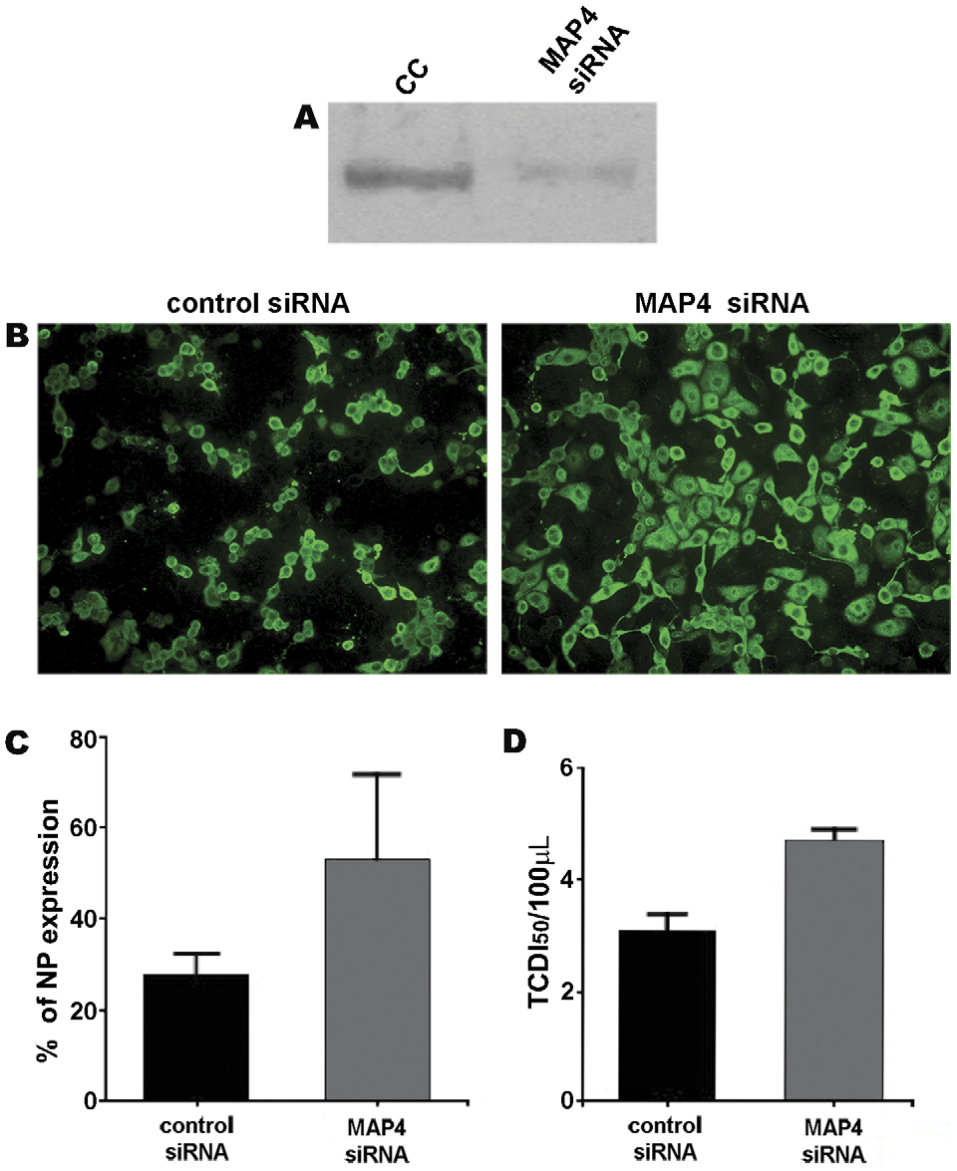
The siRNA-mediated knockdown of MAP4 gene expression improves NWS infection in LLC-MK2 cells. (A) The efficiency of MAP4 depletion was assessed by Western blotting analysis of MAP4 expression in untransfected (CC) and 48 h-transfected (MAP4 siRNA) LLC-MK2 cells. (B,C,D) LLC-MK2 cells were transfected for 48 h with control (control siRNA) or MAP4 (MAP4 siRNA) siRNAs, and then infected with NWS virus at a m.o.i. of 0.1 for 24 h. (B) IIF staining with anti-NP antibodies. Images were collected by using a conventional fluorescence microscope (magnification: 200×). (C) The percentages of cells expressing the NP antigen are shown. (D) Viral yields from culture supernatants evaluated by TCDI_50_ assays. Bars represent standard deviations. Values were means of three independent experiments.

Finding that LLC-MK2 cells possess high levels of MAP4, we were interested to investigate the effects of MAP4 depletion on NWS infection by using a RNA-mediated RNA interference approach. To this purpose, we carried out the silencing of MAP4 in LLC-MK2 cells as described in the Methods section, and then we assessed its efficiency (Figure 8A). Next, LLC-MK2 cells depleted of MAP4 were infected with NWS virus (m.o.i. = 0.1 p.f.u./cell, 24 h), before IIF analyses. Interestingly, the results in Figure 8B-C showed that NP levels were nearly doubled in LLC-MK2 cells with a knocked down expression of MAP4, when compared to control cells. Untransfected and infected LLC-MK2 cells produced IIF data (not shown) very similar to those of transfected control cells. After that, we investigated whether MAP4 depletion might improve the emergence of the viral progeny by using TCID_50_ assays. The results shown in Figure 8D were consistent with IIF analyses.

## Discussion

MTs are very stable or highly dynamic, depending on cell type, cell cycle phase, and interactions with a large number of proteins [1]–[3]. It has been suggested that post-translational modifications establish discrete MT subpopulations, which may vary among cells at different stages of polarity [3], [8], [28]. Such features make the MT cytoskeleton an excellent candidate to assist the nuclear delivery of viruses [14], [29]–[31]. Although the cellular functions of MTs have been broadly dissected, their dynamics and, very important, their impact at a subcellular level, are poorly understood.

The present study focuses on the role of MT dynamics during the initiation of NWS infection in LLC-MK2 and MDCK mammalian kidney cells.

Initial experiments assessed the efficacy of NOC and TAX in perturbing the spatial organization of the MT cytoskeleton. Then, to have a general idea on the effects of NOC and TAX on NWS replication, different time points were examined during a 24 h-infection at a low m.o.i. in kidney cells. Intriguingly, the breakdown of MTs, conversely to MT-stabilization, resulted in an enhanced infection outcome in LLC-MK2 cells. On the other hand, NWS replication in MDCK cells was barely influenced by MT-modulators. A possible explanation of these results relies on the differential infectious entry of NWS in LLC-MK2 and MDCK cells [26], seemingly implying a dissimilar dependence from the MT cytoskeleton. In this regard, an attractive hypothesis is that MT-modulators may promote the intracellular survival of NWS when clathrin-mediated endocytosis is used, such as in MDCK cells [26]. Accordingly, disruption or stabilization of MTs could arrest the transport of endosome carrier vesicles to late endosomes [32], where viral degradation takes place. It has also been shown that in MDCK cells NOC inhibits the basolateral to apical transcytosis [33], and both NOC and TAX perturb the transport of influenza hemagglutinin to the apical membrane [34]. The latter data, by highlighting the differential spatial organization of the MT cytoskeleton in polarized MDCK cells, suggest that MTs are dispensable in regulating the cytoplasmic transport from the apical to the basolateral direction, such as that occurring in this model. Thus, the rather absolute absence of drug-mediated effects on NWS replication in MDCK cells may rely on the lack of MTs intervention on virus uptake at the apical membrane. On the other hand, a quite different behaviour has been observed in LLC-MK2 cells, where the basolateral side may also be recruited [25]. Moreover, MT-independent routes playing a key role in virion transport along the infectious pathway have been described [35]–[37].

Interestingly, supplementary data obtained in the present work by using NSK cells outlined an adverse effect on NWS infection for both MT-modulators, thus supporting the notion that MTs are highly involved in this model. Nevertheless, cautious conclusions were drawn, mostly considering that a 24 h-infection allows multiple replication rounds, and thus is not straightforward to differentiate between early and late effects.

Finding that major interferences of MT-stabilization on NWS growth were detected in LLC-MK2 cells, we focused our attention on this aspect, in order to assess the timing of the aforementioned effects. To this mean, the use of a 5 h-time point, which allows a single NWS replication cycle [26], evidenced that MT-stabilization negatively influences the early phases of viral infection in LLC-MK2 cells.

It has been shown that viruses hijack MT-associated dynein motors to reach the nucleoplasm [15], [38]–[41]. In order to characterize the retrograde cytoplasmic transport of NWS in LLC-MK2 cells, we carried out 5-h infections in the presence of a dynein inhibitor. Interestingly, although we assessed that NWS uses dyneins, a percentage of NP-positive cells was still detected upon their inhibition. Thus, we assumed that NWS might alternatively recruit the actin-based motility when the MT-based trafficking is not functional. Accordingly, MT-depolymerization during the early phases of viral replication positively influences the infection outcome in LLC-MK2 cells. Indeed, we previously demonstrated the co-localization between viral NP and microfilaments during the early events of NWS infection in LLC-MK2 cells [25].

MT-isolation and high-salt extraction assays suggested the existence of interactions between viral HA and MTs in LLC-MK2 cells. Although these findings may represent a further evidence about the involvement of MTs during NWS infection in LLC-MK2 cells, our data are not conclusive and would deserve further investigation.

MT dynamics are likely to influence the rate of viral transport to the nucleus. In this regard, it has been suggested that molecular motors are able to discern post-translational modifications of tubulin [41], [42]. Moreover, post-translational acetylation of alpha-tubulin – mostly associated with stable MTs – is conceived as a key regulatory mechanism which promotes both MT-based transport and compartmentalization of subcellular organelles and signalling pathways [8], [43], [44]. In addition, post-translational acetylation directly acts on catabolic pathways and cell defence mechanisms [45]. However, opposing results were reported by Joseph *et al.* [46] who showed that tubulin acetylation impairs the endocytic trafficking.

Very important, the kinetics of alpha-tubulin acetylation upon NWS virus infection showed that mock-infected LLC-MK2 possess higher levels of acetylated MTs than MDCK cells. Moreover, although during NWS infection alpha-tubulin hyperacetylation occurred earlier in MDCK than in LLC-MK2 cells, our findings seem to exclude its involvement in the initiation of the viral life cycle in both models. Consistent with our results, Husain and Harrod [23] showed that upon infection with the New Caledonia strain of influenza A virus in MDCK cells the hyperacetylation occurs between 6 and 12 h p.i., thus implying its intervention in late phases of viral replication. In addition, it has been shown that other viruses promote MT-acetylation at late times of infection [47]–[49]. On the other hand, different conclusions were drawn by Naranatt *et al.* [31] and conflicting results reported by Warren and Cassimeris [50]. Although the contribution of tubulin hyperacetylation at different stages of viral infection is well acknowledged, here we report new insights on the capacity of NWS virus to hijack post-translational acetylation in a cell type-dependent way. Given the discrepancy in the amount of stable MTs detected in mock-infected LLC-MK2 and MDCK cells, we may speculate on the fact that NWS virus differentially activates host-dependent signalling pathways to modulate MT dynamics.

Interestingly, Gao *et al.* [51] evidenced that the MT-associated histone deacetylase 6 activity is required for both protein transport and macropinocytosis. Furthermore, Chang *et al.* [52] reported that nocodazole promotes alpha-tubulin deacetylation by activating Rho GTPase and histone deacetylase 6. In this view, we assumed that the highly acetylated MTs of mock-infected LLC-MK2 cells represent a restriction condition to the most effective NWS internalization pathway of this cellular model, the macropinocytosis, as previously described [26]. Moreover, the enhancement of NWS growth induced by nocodazole in LLC-MK2 cells might be coupled to a decreased tubulin acetylation.

It has been suggested that MAP4, the most abundant non-tubulin component of MTs in non-neuronal cells, is implicated in the cytoplasmic transport of organelles and vesicles [53]–[55] and stimulates MT-polymerization [56]. Nevertheless, conflicting results were reported by Wang *et al.* [57]. Interestingly, it has been evidenced that MAP4 overexpression inhibits vesicular transport [58] and increases both MT-polymerization and taxane sensitivity [59]. Here we show that the LLC-MK2 model has higher levels of MAP4 than MDCK cells, seemingly implying both increased MT polymer levels and interferences between molecular cargos and motors along MT tracks. Even though the observed reduction of MAP4 levels upon NWS infection in the LLC-MK2 model is highly suggestive of a host cell shut-off, a virus-induced recruitment of proteases acting against MAP4 cannot be ruled out at the present time. In this view, the cleavage of MAP4 and MAP2 has been characterized during viral infection [60], [61].

Intriguingly, here we report that viral growth in LLC-MK2 cells is significantly improved when MAP4 silencing is established at the beginning of NWS infection. Interestingly, a relationship between specific MAPs and autophagy has been described [62], [63]. Although the role of autophagy in influenza virus infection is well documented [64], [65], further experiments are needed to better elucidate the involvement of MAP4 in both viral cytoplasmic trafficking and autophagy.

In conclusion, this study contributes to improve our understanding on the differential behaviour of NWS influenza strain in two mammalian cell models. Very important, here we provide new insights into the cell type-dependent regulatory role of MT dynamics during the initiation of NWS infection. In this regard, it is likely that NWS virus get trapped when we induce MT-stabilization by drug treatments during the early stages of viral infection in LLC-MK2 but not in MDCK cells. By a molecular approach, we also demonstrate that MAP4-mediated MT-polymerization induces restriction to the initiation of NWS infection in the LLC-MK2 model.

Given that MT-stabilization represents a restriction factor for the initiation of NWS infection in LLC-MK2 cells, the present results deserve further in-depth studies in order to elucidate the intimate mechanisms that control these events.

## Supporting Information

Figure S1 **Effects of MT-modulators on both MT cytoskeleton morphology and NWS growth in NSK cells.** (A) NSK cells were either untreated (CC) or subjected to TAX or NOC treatments for 3 h 45 min, before IIF assays with anti-beta tubulin antibodies. Pictures were collected by using a conventional fluorescence microscope (magnification: 500×). Essentially similar results were obtained in two independent experiments. (B) NSK cells were mock-treated (NT) or pretreated with NOC or TAX for 30 min (0–2 h; 0–24 h), and subsequently inoculated with NWS virus (m.o.i. = 0.1 p.f.u./cell; 24 h) in the absence (NT) or presence of drugs. NOC and TAX were either kept in the culture media for 2 h p.i. and then withdrawn (0–2 h) or maintained throughout the entire infectious period (0–24 h). Alternatively, NWS infection was carried out for 5 h in drug-free culture medium, and then the cells were treated with NOC or TAX for the remnant infectious period (5–24 h). Next, the cells were labelled with anti-NP antibodies by IIF. The number of NP-positive cells in relation to total cell population was expressed as a percentage. Each sample was processed in duplicate. Values represent the mean of two independent experiments. Error bars in graphs represent standard deviations.
(TIF)

## References

[1] Downing KH (2000). Structural basis for the interaction of tubulin with proteins and drugs that affect microtubule dynamics.. Annu Rev Cell Dev Biol.

[2] Hammond JW, Cai D, Verhey KJ (2008). Tubulin modifications and their cellular functions.. Curr Opin Cell Biol.

[3] Wade RH (2009). On and around microtubules: an overview.. Mol Biotechnol.

[4] Andersen SS (2000). Spindle assembly and the art of regulating microtubule dynamics by MAPs and Stathmin/Op18.. Trends Cell Biol.

[5] Drewes G, Ebneth A, Mandelkow EM (1998). MAPs, MARKs and microtubule dynamics.. Trends Biochem Sci.

[6] Balasubramani M, Nakao C, Uechi GT, Cardamone J, Kamath K (2011). Characterization and detection of cellular and proteomic alterations in stable stathmin-overexpressing, taxol-resistant BT549 breast cancer cells using offgel IEF/PAGE difference gel electrophoresis.. Mutat Res.

[7] Howell B, Larsson N, Gullberg M, Cassimeris L (1999). Dissociation of the tubulin-sequestering and microtubule catastrophe-promoting activities of oncoprotein 18/stathmin.. Mol Biol Cell.

[8] Perdiz D, Mackeh R, Poüs C, Baillet A (2011). The ins and outs of tubulin acetylation: more than just a post-translational modification?. Cell Signal.

[9] Westermann S, Weber K (2003). Post-translational modifications regulate microtubule function.. Nat Rev Mol Cell Biol.

[10] Matsuyama A, Shimazu T, Sumida Y, Saito A, Yoshimatsu Y (2002). In vivo destabilization of dynamic microtubules by HDAC6-mediated deacetylation.. EMBO J.

[11] Gross SP, Vershinin M, Shubeita GT (2007). Cargo transport: two motors are sometimes better than one.. Curr Biol.

[12] Welte MA (2010). Bidirectional transport: matchmaking for motors.. Curr Biol.

[13] Döhner K, Nagel CH, Sodeik B (2005). Viral stop-and-go along microtubules: taking a ride with dynein and kinesins.. Trends Microbiol.

[14] Radtke K, Döhner K, Sodeik B (2006). Viral interactions with the cytoskeleton: a hitchhiker's guide to the cell.. Cell Microbiol.

[15] Smith GA, Enquist LW (2002). Break ins and break outs: viral interactions with the cytoskeleton of mammalian cells.. Annu Rev Cell Dev Biol.

[16] Cantin R, Méthot S, Tremblay MJ (2005). Plunder and stowaways: incorporation of cellular proteins by enveloped viruses.. J Virol.

[17] Johannsen E, Luftig M, Chase MR, Weicksel S, Cahir-McFarland E (2004). Proteins of purified Epstein-Barr virus.. Proc Natl Acad Sci U S A.

[18] Shaw ML, Stone KL, Colangelo CM, Gulcicek EE, Palese P (2008). Cellular proteins in influenza virus particles.. PLoS Pathog.

[19] Momose F, Kikuchi Y, Komase K, Morikawa Y (2007). Visualization of microtubule-mediated transport of influenza viral progeny ribonucleoprotein.. Microbes Infect.

[20] Fislová T, Thomas B, Graef KM, Fodor E (2010). Association of the influenza virus RNA polymerase subunit PB2 with the host chaperonin CCT.. J Virol.

[21] Amorim MJ, Bruce EA, Read EK, Foeglein A, Mahen R (2011). A rab11- and microtubule-dependent mechanism for cytoplasmic transport of influenza A virus viral RNA.. J Virol.

[22] Momose F, Sekimoto T, Ohkura T, Jo S, Kawaguchi A (2011). Apical transport of influenza A virus ribonucleoprotein requires Rab11-positive recycling endosome.. PLoS One.

[23] Husain M, Harrod KS (2011). Enhanced acetylation of alpha-tubulin in influenza A virus infected epithelial cells.. FEBS Lett.

[24] Yamauchi Y, Boukari H, Banerjee I, Sbalzarini IF, Horvath P (2011). Histone deacetylase 8 is required for centrosome cohesion and influenza A virus entry.. PLoS Pathog.

[25] Arcangeletti MC, De Conto F, Ferraglia F, Pinardi F, Gatti R (2008). Host-cell-dependent role of actin cytoskeleton during the replication of a human strain of influenza A virus.. Arch Virol.

[26] De Conto F, Covan S, Arcangeletti MC, Orlandini G, Gatti R (2011). Differential infectious entry of human influenza A/NWS/33 virus (H1N1) in mammalian kidney cells.. Virus Res.

[27] Kannan H, Fan S, Patel D, Bossis I, Zhang YJ (2009). The hepatitis E virus open reading frame 3 product interacts with microtubules and interferes with their dynamics.. J Virol.

[28] Quinones GB, Danowski BA, Devaraj A, Singh V, Ligon LA (2011). The posttranslational modification of tubulin undergoes a switch from detyrosination to acetylation as epithelial cells become polarized.. Mol Biol Cell.

[29] Alonso C, Miskin J, Hernáez B, Fernandez-Zapatero P, Soto L (2001). African swine fever virus protein p54 interacts with the microtubular motor complex through direct binding to light-chain dynein.. J Virol.

[30] Gilbert JM, Goldberg IG, Benjamin TL (2003). Cell penetration and trafficking of polyomavirus.. J Virol.

[31] Naranatt PP, Krishnan HH, Smith MS, Chandran B (2005). Kaposi's sarcoma-associated herpesvirus modulates microtubule dynamics via RhoA-GTP-diaphanous 2 signaling and utilizes the dynein motors to deliver its DNA to the nucleus.. J Virol.

[32] Apodaca G (2001). Endocytic traffic in polarized epithelial cells: role of the actin and microtubule cytoskeleton.. Traffic.

[33] Hunziker W, Mâle P, Mellman I (1990). Differential microtubule requirements for transcytosis in MDCK cells.. EMBO J.

[34] van Zeijl MJ, Matlin KS (1990). Microtubule perturbation inhibits intracellular transport of an apical membrane glycoprotein in a substrate-dependent manner in polarized Madin-Darby canine kidney epithelial cells.. Cell Regul.

[35] Glotzer JB, Michou AI, Baker A, Saltik M, Cotten M (2001). Microtubule-independent motility and nuclear targeting of adenoviruses with fluorescently labeled genomes.. J Virol.

[36] Yoder A, Guo J, Yu D, Cui Z, Zhang XE (2011). Effects of microtubule modulators on HIV-1 infection of transformed and resting CD4 T cells.. J Virol.

[37] Vaughan JC, Brandenburg B, Hogle JM, Zhuang X (2009). Rapid actin-dependent viral motility in live cells.. Biophys J.

[38] Frampton AR, Uchida H, von Einem J, Goins WF, Grandi P (2010). Equine herpesvirus type 1 (EHV-1) utilizes microtubules, dynein, and ROCK1 to productively infect cells.. Vet Microbiol.

[39] Lakadamyali M, Rust MJ, Babcock HP, Zhuang X (2003). Visualizing infection of individual influenza viruses.. Proc Natl Acad Sci U S A.

[40] Seisenberger G, Ried MU, Endress T, Büning H, Hallek M (2001). Real-time single-molecule imaging of the infection pathway of an adeno-associated virus.. Science.

[41] Suikkanen S, Aaltonen T, Nevalainen M, Välilehto O, Lindholm L (2003). Exploitation of microtubule cytoskeleton and dynein during parvoviral traffic toward the nucleus.. J Virol.

[42] Cai D, McEwen DP, Martens JR, Meyhofer E, Verhey KJ (2009). Single molecule imaging reveals differences in microtubule track selection between kinesin motors.. PLoS Biol.

[43] Reed NA, Cai D, Blasius TL, Jih GT, Meyhofer E (2006). Microtubule acetylation promotes kinesin-1 binding and transport.. Curr Biol.

[44] Gao YS, Hubbert CC, Yao TP (2010). The microtubule-associated histone deacetylase 6 (HDAC6) regulates epidermal growth factor receptor (EGFR) endocytic trafficking and degradation.. J Biol Chem.

[45] Xie R, Nguyen S, McKeehan WL, Liu L (2010). Acetylated microtubules are required for fusion of autophagosomes with lysosomes.. BMC Cell Biol.

[46] Joseph RA, Shepard BD, Kannarkat GT, Rutledge TM, Tuma DJ (2008). Microtubule acetylation and stability may explain alcohol-induced alterations in hepatic protein trafficking.. Hepatology.

[47] Parker JS, Broering TJ, Kim J, Higgins DE, Nibert ML (2002). Reovirus core protein mu2 determines the filamentous morphology of viral inclusion bodies by interacting with and stabilizing microtubules.. J Virol.

[48] Jouvenet N, Monaghan P, Way M, Wileman T (2004). Transport of African swine fever virus from assembly sites to the plasma membrane is dependent on microtubules and conventional kinesin.. J Virol.

[49] Yedowitz JC, Kotsakis A, Schlegel EF, Blaho JA (2005). Nuclear localizations of the herpes simplex virus type 1 tegument proteins VP13/14, vhs, and VP16 precede VP22-dependent microtubule reorganization and VP22 nuclear import.. J Virol.

[50] Warren JC, Cassimeris L (2007). The contributions of microtubule stability and dynamic instability to adenovirus nuclear localization efficiency.. Cell Motil Cytoskeleton.

[51] Gao YS, Hubbert CC, Lu J, Lee YS, Lee JY (2007). Histone deacetylase 6 regulates growth factor-induced actin remodeling and endocytosis.. Mol Cell Biol.

[52] Chang YC, Nalbant P, Birkenfeld J, Chang ZF, Bokoch GM (2008). GEF-H1 couples nocodazole-induced microtubule disassembly to cell contractility via RhoA.. Mol Biol Cell.

[53] Bulinski JC, Borisy GG (1980). Widespread distribution of a 210,000 mol wt microtubule-associated protein in cells and tissues of primates.. J Cell Biol.

[54] Holmfeldt P, Brattsand G, Gullberg M (2002). MAP4 counteracts microtubule catastrophe promotion but not tubulin-sequestering activity in intact cells.. Curr Biol.

[55] Parysek LM, Wolosewick JJ, Olmsted JB (1984). MAP 4: a microtubule-associated protein specific for a subset of tissue microtubules.. J Cell Biol.

[56] Mandelkow E, Mandelkow EM (1995). Microtubules and microtubule-associated proteins.. Curr Opin Cell Biol.

[57] Wang XM, Peloquin JG, Zhai Y, Bulinski JC, Borisy GG (1996). Removal of MAP4 from microtubules in vivo produces no observable phenotype at the cellular level.. J Cell Biol.

[58] Bulinski JC, McGraw TE, Gruber D, Nguyen HL, Sheetz MP (1997). Overexpression of MAP4 inhibits organelle motility and trafficking in vivo.. J Cell Sci.

[59] Hait WN, Yang JM (2006). The individualization of cancer therapy: the unexpected role of p53.. Trans Am Clin Climatol Assoc.

[60] Joachims M, Harris KS, Etchison D (1995). Poliovirus protease 3C mediates cleavage of microtubule-associated protein 4.. Virology.

[61] Aprea S, Del Valle L, Mameli G, Sawaya BE, Khalili K (2006). Tubulin-mediated binding of human immunodeficiency virus-1 Tat to the cytoskeleton causes proteasomal-dependent degradation of microtubule-associated protein 2 and neuronal damage.. J Neurosci.

[62] Xie R, Nguyen S, McKeehan K, Wang F, McKeehan WL (2011). Microtubule-associated protein 1S (MAP1S) bridges autophagic components with microtubules and mitochondria to affect autophagosomal biogenesis and degradation.. J Biol Chem.

[63] Wang QJ, Ding Y, Kohtz DS, Mizushima N, Cristea IM (2006). Induction of autophagy in axonal dystrophy and degeneration.. J Neurosci.

[64] Noda T, Fujita N, Yoshimori T (2009). The late stages of autophagy: how does the end begin?. Cell Death Differ.

[65] Zhou Z, Jiang X, Liu D, Fan Z, Hu X (2009). Autophagy is involved in influenza A virus replication.. Autophagy.

