# Supplementary figures and images for: Highly Dynamic Microtubules Improve the Effectiveness of Early Stages of Human Influenza A/NWS/33 Virus Infection in LLC-MK2 Cells
Source: PLoS One. 2012 Jul 20;7(7):e41207. doi: 10.1371/journal.pone.0041207 (PMC3401105; doi:10.1371/journal.pone.0041207)

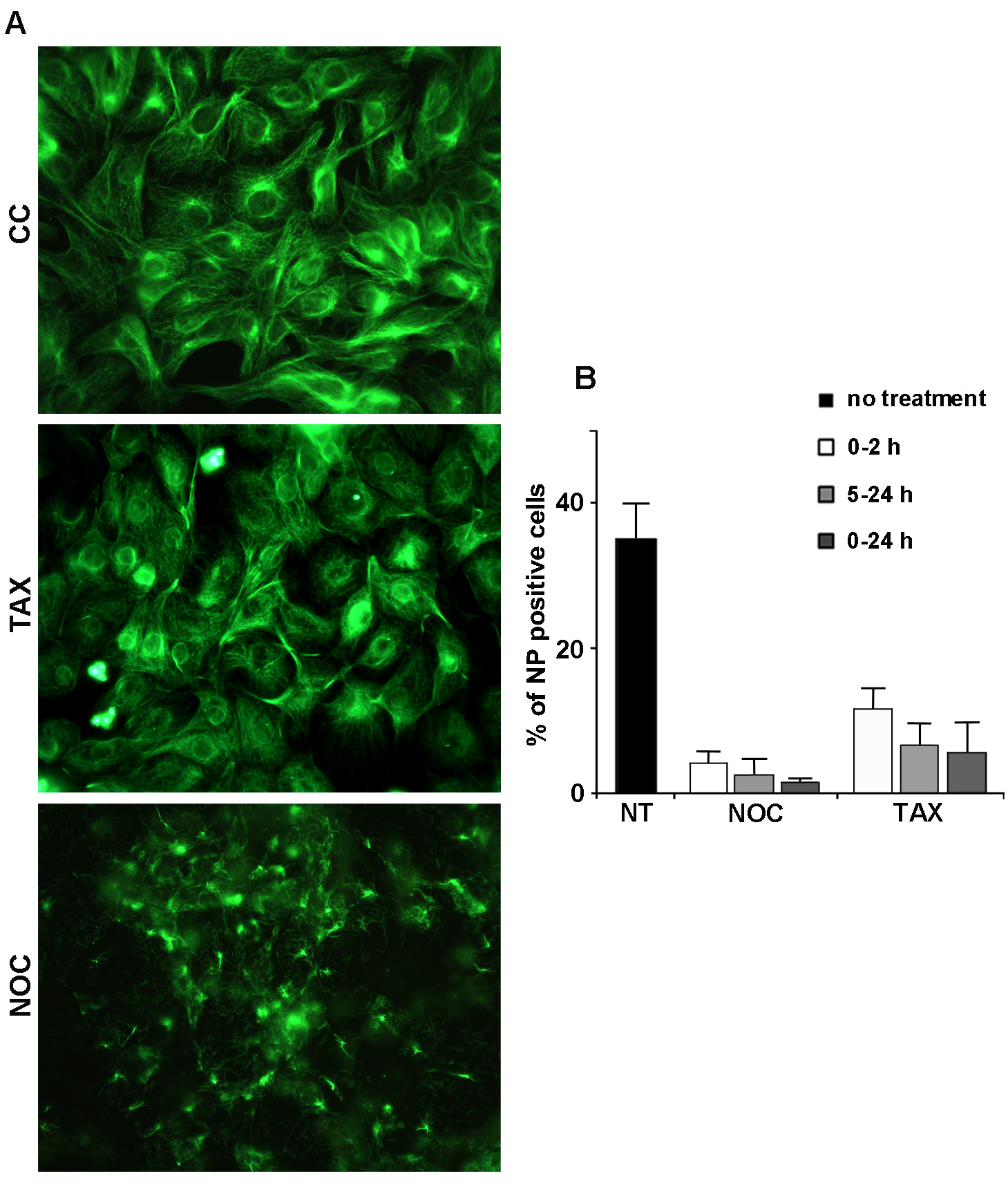

Supplement: Figure S1 — Effects of MT-modulators on both MT cytoskeleton morphology and NWS growth in NSK cells. (A) NSK cells were either untreated (CC) or subjected to TAX or NOC treatments for 3 h 45 min, before IIF assays with anti-beta tubulin antibodies. Pictures were collected by using a conventional fluorescence microscope (magnification: 500×). Essentially similar results were obtained in two independent experiments. (B) NSK cells were mock-treated (NT) or pretreated with NOC or TAX for 30 min (0–2 h; 0–24 h), and subsequently inoculated with NWS virus (m.o.i. = 0.1 p.f.u./cell; 24 h) in the absence (NT) or presence of drugs. NOC and TAX were either kept in the culture media for 2 h p.i. and then withdrawn (0–2 h) or maintained throughout the entire infectious period (0–24 h). Alternatively, NWS infection was carried out for 5 h in drug-free culture medium, and then the cells were treated with NOC or TAX for the remnant infectious period (5–24 h). Next, the cells were labelled with anti-NP antibodies by IIF. The number of NP-positive cells in relation to total cell population was expressed as a percentage. Each sample was processed in duplicate. Values represent the mean of two independent experiments. Error bars in graphs represent standard deviations. (TIF) [file pone.0041207.s001.tif]
